# Advances and Mechanisms of RNA–Ligand Interaction Predictions

**DOI:** 10.3390/life15010104

**Published:** 2025-01-15

**Authors:** Chen Zhuo, Chengwei Zeng, Haoquan Liu, Huiwen Wang, Yunhui Peng, Yunjie Zhao

**Affiliations:** 1 Institute of Biophysics and Department of Physics, Central China Normal University, Wuhan 430079, China; 2 School of Physics and Engineering, Henan University of Science and Technology, Luoyang 471023, China; huiwenwang@haust.edu.cn

**Keywords:** RNA secondary structure motifs, RNA pocket geometric feature, RNA–ligand interaction mechanism, structure prediction

## Abstract

The diversity and complexity of RNA include sequence, secondary structure, and tertiary structure characteristics. These elements are crucial for RNA’s specific recognition of other molecules. With advancements in biotechnology, RNA–ligand structures allow researchers to utilize experimental data to uncover the mechanisms of complex interactions. However, determining the structures of these complexes experimentally can be technically challenging and often results in low-resolution data. Many machine learning computational approaches have recently emerged to learn multiscale-level RNA features to predict the interactions. Predicting interactions remains an unexplored area. Therefore, studying RNA–ligand interactions is essential for understanding biological processes. In this review, we analyze the interaction characteristics of RNA–ligand complexes by examining RNA’s sequence, secondary structure, and tertiary structure. Our goal is to clarify how RNA specifically recognizes ligands. Additionally, we systematically discuss advancements in computational methods for predicting interactions and to guide future research directions. We aim to inspire the creation of more reliable RNA–ligand interaction prediction tools.

## 1. Introduction

RNA often interacts with other molecules to carry out its biological functions [1,2,3]. Ligands are crucial in regulating RNA functions, affecting RNA activity and stability through various interactions [4]. For instance, benzimidazole translation inhibitors bind to the internal ribosome entry site of the hepatitis C virus, which leads to significant conformational changes of a deep pocket in the RNA [5]. The glmS riboswitch interacts with the metabolite glucosamine 6-phosphate (GlcN6P), a product of the synthase reaction, which acts as a cofactor for self-cleavage. This process regulates metabolite levels and inhibits bacterial cell wall synthesis [6]. Therefore, exploring RNA–ligand interactions is essential to uncovering the functional mechanisms of RNA and understanding its role in biological processes.

The diversity and complexity of the RNA structure enable it to recognize various molecules effectively. Research on the dynamic conformations of riboswitches has identified two primary modes of RNA–ligand interactions: conformational selection and induced fit [7,8,9,10]. For instance, the free SAM-II riboswitch initially forms a simple hairpin structure without the ligand [11]. However, when the SAM ligand is first recognized, the riboswitch undergoes local conformational changes that allow it to stabilize and create a suitable binding site for the ligand. This demonstrates that RNA exhibits significant versatility and plasticity, allowing it to fold into various three-dimensional (3D) shapes that provide specific binding sites for different molecules. With the advancement of biotechnology, an increasing number of RNA-related databases have been established [12,13]. For example, the SMMRNA and NALDB databases provide detailed information on RNA–ligand experimental data, including RNA sequences and structural information, as well as the chemical information of ligands and their binding affinities (Ki, Kd, IC50) for these interactions [14,15]. Other databases, such as ncRPheno, ViRBase v3.0, and LncRNADisease v3.0, catalog the associations between miRNAs, lncRNAs, and other non-coding RNAs, and various diseases in humans and other mammals [16,17,18]. These data sources lay a solid foundation for studying RNA interactions, allowing researchers to explore the interaction interfaces between RNA and their binding partners visually. However, experimental methods such as X-ray crystallography, NMR, and cryo-electron microscopy present several challenges, including high costs and operational complexity [19,20,21,22]. As of 27 November 2024, the Protein Data Bank contains 227,561 experimental structures, but fewer than 1000 of them are RNA–ligand structures [23]. Therefore, there is an urgent need to develop computational methods for predicting RNA interactions.

Machine-learning-based computational methods have recently become essential tools for understanding RNA structure and function [24,25,26]. By leveraging prior knowledge from experimental structures, these methods can analyze vast biological datasets, identify complex patterns, and predict interactions that were previously difficult to detect experimentally [27,28,29]. For instance, RNet utilizes RNA structure network features from three machine learning models to predict RNA–small molecule binding sites [30]. On the other hand, RNAsite employs a random forest model to learn RNA sequence and structure features at various levels to predict RNA–ligand binding sites [31]. Furthermore, deep-learning-based computational methods are emerging as powerful tools for predicting RNA interactions [32,33]. ZHmolReSTasite, for example, uses a deep residual network model to systematically learn RNA sequence, secondary structure, and tertiary structure features for predicting ligand-binding nucleotides [34]. MultiModRLBP combines four deep learning modules to capture multidimensional RNA structural features and predict these nucleotides [35]. However, current methods for predicting RNA–ligand binding affinity are limited, with only RLaffinity and RSAPred available [36,37]. Despite being a relatively unexplored area in RNA research, machine learning algorithms show tremendous potential. RNA structural features and interaction information with other molecules provide valuable data resources and theoretical guidance for computational methods, forming a solid foundation for model training.

**Figure 1:**
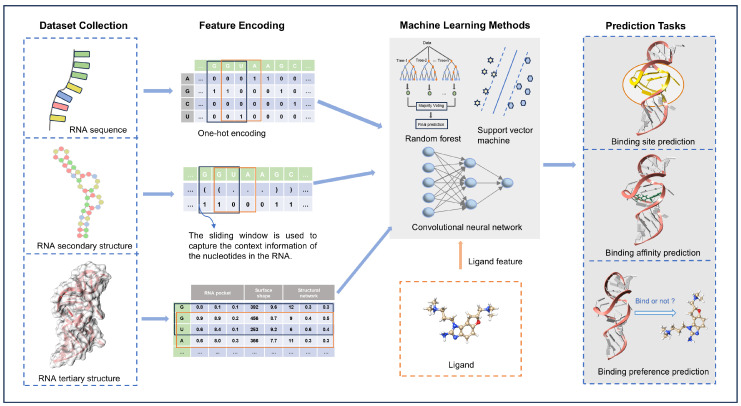
RNA features at different levels are used to predict RNA–ligand interactions, including sequence, secondary structure, and tertiary structure features.

This review provides a thorough analysis of RNA–ligand interactions, focusing on the mechanisms through which RNA recognizes ligands and their applications in machine learning algorithms (Figure 1). To aid researchers in understanding the biological functions of RNA, we systematically compile representative databases of RNA–ligand interactions. From the perspective of RNA, we explore how it specifically recognizes ligands through physicochemical interactions across various levels, including primary sequence, secondary structure, and tertiary structure. This approach allows us to gain deeper insights into the mechanisms of RNA recognition. Additionally, we analyze the progress and challenges in computational methods for predicting RNA–ligand interactions, considering different aspects of RNA features. We aim to provide a comprehensive overview of the potential development of computational tools for exploring RNA–ligand interactions, emphasizing the analysis of interaction mechanisms that involve multiple dimensions of RNA characteristics.

## 2. RNA–Ligand Database Resources

**Figure 2:**
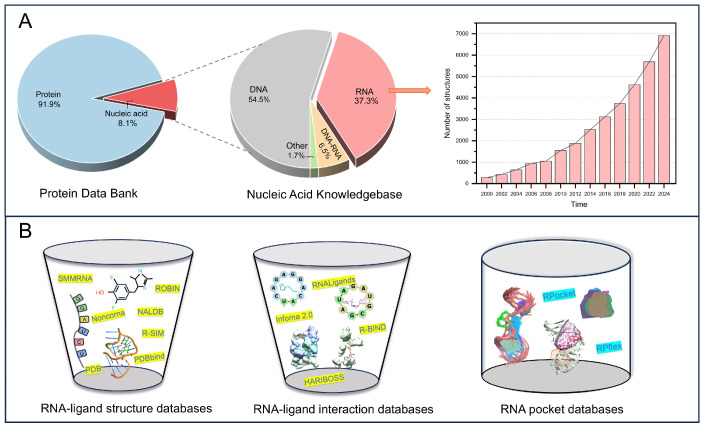
(**A**) The structure distribution of PDB and NAKB databases, and the number of NAKB database structures over time. (**B**) The type distribution of RNA–ligand databases, including structure, interaction, and RNA pocket databases.

The Protein Data Bank (PDB) is a comprehensive database that stores and retrieves experimental structural information for biological macromolecules [23]. Currently, approximately 92% of the entries in the PDB are protein-related, while around 8% are pertaining to nucleic acids (Figure 2A). The Nucleic Acid Knowledgebase (NAKB) aggregates all RNA and DNA experimental structures [38]. It provides external resource lists and various interactive tools. As of 27 November 2024, the NAKB offers 18,645 available structures, with RNA complexes making up 37% of these, translating to 6914 entries. However, there are fewer than 1000 RNA–ligand complexes. In recent years, advancements have been made in techniques for resolving RNA complex structures, enabling researchers to uncover the structural details of these complexes at the atomic level [19]. Nevertheless, experimentally elucidating the complexity and dynamics of RNA structures, as well as their responses to environmental changes, remains a formidable challenge. Despite this, the structural data in databases like the PDB and NAKB provide a solid foundation for analyzing and understanding the critical structural features and functional roles of RNA–ligand interactions.

RNA–ligand databases are essential for studying RNA’s biological functions (Table 1). These databases can be divided into two categories: structural databases and interaction databases (Figure 2B). RNA–ligand structural databases provide experimentally determined information about complexes, including RNA sequences and structures, ligand molecular properties, and binding thermodynamic data. For instance, NoncoRNA compiles experimentally validated interactions between non-coding RNAs and drug targets, featuring 8233 entries comprising 5568 ncRNAs and 154 drugs across 134 cancer types [39]. ROBIN is a novel library of nucleic acid binders that reports comprehensive results from small-molecule microarray screenings targeting 36 individual nucleic acids with 24,572 small molecules [40]. Other SMMRNA, NALDB, PDBbind, and R-SIM databases provide experimentally determined RNA–ligand structures and thermodynamic data, including binding affinities, binding free energies, and experimental conditions [14,15,41,42]. RNA–ligand interaction databases provide detailed information about the interaction interfaces between RNA and ligands. This includes the sequence and structure of the binding interface and the physicochemical environment. For example, RNALigands integrates RNA–ligand interaction data from Inforna 2.0, R-BIND, and the PDB database, offering sequence and structural data for 841 RNA secondary structure motif–ligand pairs [43,44,45]. RNALigands suggests that similar secondary structure motifs tend to bind to similar ligands, indicating that RNA’s secondary structural features carry important information for specific ligand recognition. HARIBOSS analyzes RNA–ligand structural interaction data from the PDB database, providing annotations on the physicochemical properties of ligands and the structures and compositions of RNA pockets at the binding interface [46]. HARIBOSS found that RNA pockets exhibit hydrogen bond donor and acceptor properties similar to those present in proteins. In addition, druggable RNA pockets tend to be more hydrophobic and less exposed to solvents, underscoring the significance of the RNA structural surface shape in ligand recognition.

RNA–ligand databases are essential resources for understanding RNA function and advancing computational methods. However, RNA demonstrates significant dynamics in recognizing other molecules, with multiple conformational states being fundamental to its function [22,48]. While these databases provide experimental structural data and static interaction information, they do not adequately explore or analyze the 3D structural flexibility and geometric features. To address this gap, we developed the RNA pocket databases (RPocket and RPflex), which capture the static and dynamic interactions of RNA–ligand complexes by examining the geometric features and conformational changes of RNA pockets (Figure 2B). The RPocket database focuses on static information related to RNA sequences and structures, calculating geometric properties of ligand-binding and non-binding pockets, including volume, surface area, effective radius, and centroid [47]. We also created pocket-shape descriptors, RPDescriptors, for calculating the geometric features of RNA pockets. RPDescriptor calculates the shape similarity scores, categorizing pockets into sphere-like, rod-like, and disc-like shapes. The shape similarity scores are defined by two descriptors (${rpd}_{1}$ and ${rpd}_{2}$) based on normalized principal moments of inertia ratios. Our findings indicate that the shape and geometric characteristics of pockets exhibit selective specificity for ligand binding. In addition, we assessed RNA flexibility through the conformational changes in RNA pockets, thereby capturing the dynamic features associated with RNA–ligand recognition [49]. RPflex compiled pocket datasets from various sources, including 2276 pockets from RNA complexes, 352 pockets from RNA–ligand complexes, and 526 pockets from RNA–protein complexes. However, due to the limited number of NMR experimental structures available, we collected pocket data from only 160 non-redundant RNA-related structures. To overcome the limitations of experimental data, we need to understand the physics-based foundations of interactions.

## 3. Physics-Based Interaction Forces on RNA–Ligand Complexes

RNA–ligand complex stability is achieved through interactions between nucleotides and ligands based on physical principles. These interactions include hydrogen bonds, van der Waals (VdW) forces, stacking interactions, and hydrophobic interactions, all of which are considered short-range forces. In contrast, electrostatic forces are long-range and can act with long distances. Long-range forces primarily drive the remote recognition between RNA and ligands, while short-range forces serve to optimize and enhance the structure of the complex [47].

### 3.1. The Short-Range Forces

**Figure 3:**
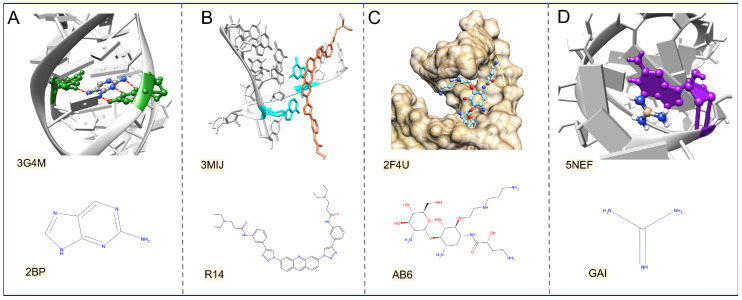
(**A**) Hydrogen bonding patterns induced by the ligand (2BP) to interact with nucleobases in the crystal structure of guanine riboswitch (PDB ID: 3G4M). (**B**) Stacking patterns induced by the ligand (R14) to interact with a telomeric RNA G-quadruplex (PDB ID:3MIJ). (**C**) The hydrophobicity-driven contacts induced by the ligand (AB6) binding to the ribosomal aminoacyl tRNA (PDB ID: 2FU4). (**D**) The cation–π interaction formed between the ligand (GAI) and the guanidine II riboswitch (PDB ID: 5NEF).

The hydrogen bond is an interaction force that occurs between a hydrogen atom and two electronegative atoms, such as oxygen and nitrogen. Each hydrogen bond typically contributes between 0.5 and 4.5 kcal/mol to the overall system energy [50]. The weakest hydrogen bonds are often considered to be van der Waals (VdW) contacts, which contribute between 0.5 and 1 kcal/mol. The detection tool, HBplus, uses default parameters for identifying hydrogen bonds: a hydrogen–acceptor distance less than 2.7 Å and a donor–acceptor distance less than 3.35 Å [51]. For VdW contacts, the default parameters define these contacts as occurring between atoms that are not involved in hydrogen bonds and with less than 3.9 Å. Hydrogen bonds are generally associated with the interaction energy between actual hydrogen bond donors and acceptors. This interaction energy can be described using the following Lennard–Jones potential [52]:

(1) $$V\left(r\right)=4\varepsilon ((\frac{\sigma }{r}{)}^{12}-{\left(\frac{\sigma }{r}\right)}^{6})$$

where ε represents the potential well depth, r represents the distance between two particles, and σ is the distance between the two particles when the potential energy is exactly zero. The bases and backbone of RNA (phosphate and ribose) can form hydrogen bonds and VdW contacts with ligands (Figure 3A) [53,54]. RPflex analyzed the interactions in ligand-binding pockets from NMR RNA–ligand structures [49]. The results show that hydrogen bonds with the bases, phosphate, and ribose account for 63%, 21%, and 16%, respectively. In contrast, VdW contacts with these components account for 72% with bases, 6% with phosphate, and 22% with ribose. These findings are consistent with previous studies, including one by G. Padroni et al., which demonstrated that hydrogen bonding events involving bases are approximately three times more frequent than those with the phosphate-ribose backbone [55]. This further reinforces the idea that bases carry most of the structural information in the RNA binding pocket and serve as the primary source of interactions with ligands.

Stacking interactions are non-covalent attractive forces between aromatic rings, contributing approximately 2–6 kcal/mol for each interaction [56]. They typically occur at interatomic distances of 2.7–4.3 Å. A typical example is the acridine-based ligand that targets a telomeric RNA G-quadruplex (Figure 3B), where the binding event is primarily driven by stacking interactions [57]. G. Padroni et al. discovered that stacking and hydrogen bond interactions represent the largest interactions in RNA–ligand complexes, accounting for 34.8% and 34.4% [55]. This highlights the significance of hydrogen bonds and stacking interactions in RNA recognition. Hydrophobic interactions generally occur at distances between 3.8 and 5.0 Å, with each interaction contributing approximately 1–2 kcal/mol [58,59]. When hydrophobic groups cluster, the surrounding water molecules increase the system’s entropy. This increase in entropy is the main driving force behind hydrophobic interactions. Some RNA-targeted drugs, such as cryptolepine hydrate and aminoglycosides, utilize hydrophobic interactions to enhance binding to their RNA targets [60,61]. As illustrated in Figure 3C, the increased affinity of an aminoglycoside analog is mainly attributed to the VdW and hydrophobic components of the solvation-free energy, which arise from the methylene chain aligning with or pointing down the major groove [62].

### 3.2. The Long-Range Force

Electrostatic interactions are a type of non-covalent force between two charged particles. Their strength depends on the quantity of the charges and the distance separating them [63]. According to Coulomb’s law, the value of electrostatic force can be calculated as follows [64]:

(2) $$F=k\frac{q_{1}q_{2}}{r^{2}}$$

where r is the distance between charge particles $q_{1}$ and $q_{2}$, and k is the Coulomb constant. There are N point charge particles distributed in a uniform dielectric, and their electrostatic potential at position R in space is calculated by

(3) $$\phi \left(r\right)=k\sum_{i}\frac{q_{i}}{\left|R-r_{i}\right|}$$

where $q_{i}$ and $r_{i}$ represent the charge and position of the particle. Electrostatic interactions occur between the negatively charged RNA phosphate backbone and positively charged ligands. Cation–π interactions are electrostatic interactions between positively charged atoms and the negatively charged electron cloud of aromatic systems [65]. A notable example of cation–π interactions in RNA recognition is guanidine binding to the guanidine II riboswitch [66]. As illustrated in Figure 3D, guanidine cations stack upon the nucleobase guanine, forming a cation–π interaction. These electrostatic forces are characterized by their long-range and non-specific nature. They play a crucial role in the initial stages of complex formation by facilitating contact and recognition between molecules. The RPocket database systematically analyzed 240 pockets in RNA–ligand complexes and proposed a potential mechanism for complex formation [47]. This mechanism suggests that long-range electrostatic interactions guide the initial recognition and binding of RNA and its ligand, while short-range interactions optimize and stabilize the resulting complexes. These interaction pairs can be regarded as distance constraints that aid in complexes’ structural modeling and drug design. For example, a nanoassembly system composed of doxorubicin-conjugated polyphosphoester and CD47-targeting siRNA utilizes these principles [67]. In this system, electrostatic interactions and π-π stacking work together to achieve targeted drug delivery, successfully reactivating T cell- and macrophage-mediated anticancer immunotherapy.

## 4. Interaction Mechanisms Extracted from RNA Features

**Figure 4:**
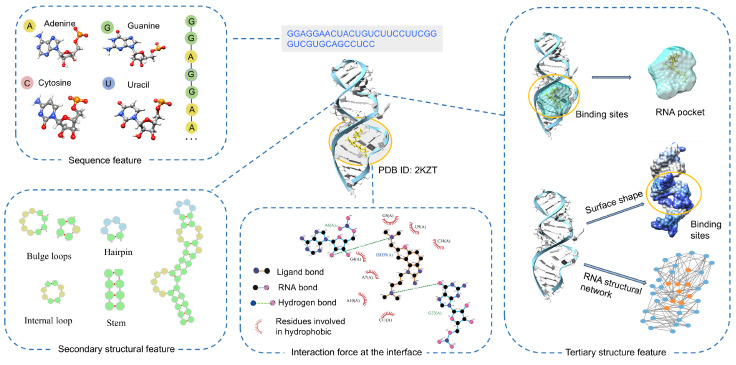
RNA structure is organized into three hierarchical levels: sequence, secondary structure, and tertiary structure. RNA features encompass three levels: sequence, secondary structure, and tertiary structure. Various physical interaction forces contribute to stabilizing the complex structures formed between RNA and other molecules, such as 2KZT.

RNA structure is organized into three hierarchical levels: sequence, secondary, and tertiary structure (Figure 4). The primary sequence determines its coding and functional properties, while its secondary and tertiary structures give rise to unique spatial configurations that enable binding to target molecules. At the binding interface, various non-covalent interactions between RNA and ligands facilitate the recognition and stabilization of the complex structure. Our aim is to investigate the interaction mechanisms that provide valuable insights for developing computational methods.

### 4.1. Sequence Features

The primary sequence of RNA refers to the linear arrangement of its four nucleotides: adenine (A), guanine (G), cytosine (C), and uracil (U). In the context of RNA–ligand interactions, most hydrogen bonds are formed with the negatively charged oxygen atoms of the phosphate groups in the RNA sugar-phosphate backbone [54,68]. However, ligands preferentially form hydrogen bonds with the nucleobases, with G being the most involved, followed by U, C, and A. Most hydrophobic interactions occur through the surfaces of the nucleobases, with minimal contact made with the sugar moiety [69]. Previous studies have indicated that optimizing stacking interactions may target purine-rich RNA motifs more effectively [69,70]. Additionally, the RPocket database has analyzed nucleotide sequence patterns derived from RNA–ligand binding sites, revealing that certain sequences, specifically “GU” (11.7%), “GG” (8.8%), “GA” (8.8%), and “GC” (8.1%), are more likely to bind with ligands [47]. Furthermore, it has been suggested that RNA sequences are more susceptible to recognition by certain small molecules. An example of this is myotonic dystrophy (DM), which is caused by repeated expansions of RNAs (CCUG) [71]. This condition leads to toxicity by sequestering crucial RNA-binding proteins and generating toxic proteins through repeat-associated non-ATG translation. Compounds developed by Childs-Disney et al., such as K-alkyne, 2K-4, and 3K-4, have been studied for their potential to improve the BIN1 pre-mRNA splicing defect in a cellular model of DM2 [72].

### 4.2. Secondary Structure Features

RNA secondary structure consists of paired and unpaired nucleotides, resulting in structural elements such as helical stems and single-stranded regions. The single-stranded regions comprise unpaired nucleotides, including hairpin loops, internal loops, bulge loops, multibranch loops, and pseudoknots [73]. In our previous work, RBind discovered that 98% of nucleotides bound by ligands are located within or near loop regions (within five nucleotide pairs of the loop regions) [74]. This suggests that loops are potential binding sites for ligands. Further analysis by RPocket examined the distribution of RNA secondary structure at ligand-binding sites [47]. The results indicated that these sites tend to be within specific loop motifs, highlighting the important role of unpaired nucleotides in ligand binding. By calculating pocket shape descriptors, we found that 92.6% of tandem loops are typically located within pockets of the same shape. Binding to incorrect secondary structure motifs may disrupt interactions and undermine structural stability. Our findings suggest that incorporating more complex loop combinations could improve predictions of RNA tertiary structures. Additionally, Disney and colleagues have shown that their RNA loop motif–ligand interaction database, Inforna 2.0, can identify ligands for targets such as microRNAs and viral RNAs [44]. Similarly, the R-BIND and RLigands databases curate pairs of loop motif–ligand interactions, allowing users to screen potential ligands by assessing secondary structure motif similarity among RNAs [43,45]. Therefore, the interactions of nucleotides within RNA secondary structures give rise to loop motifs with ligand recognition capabilities, making these motifs ideal candidates for ligand binding.

### 4.3. Tertiary Structure Features

Secondary structural elements interact spatially to form pockets in RNA. These RNA pockets are defined as concave areas on the three-dimensional structural surface, serving as specific binding sites for other molecules. Various methods have been developed to identify RNA pockets by analyzing their geometric features, such as volume, surface area, and sphericity [75,76,77]. Our previous findings indicate that most ligand-binding pockets have larger volumes and surface areas compared to non-binding pockets, suggesting that ligands might influence the motions of these pockets [47].

Scientists have identified base recognition and shape complementarity at interfaces as key factors in RNA complex interactions [78,79]. In this context, the solvent-accessible surface area (SASA) and Laplacian norm (LN) help characterize the pocket’s shape, while structural network features illustrate communication between nucleotides. Our previous work, ZHmolReSTasite, analyzed the SASA of ligand-binding and non-binding nucleotides in 60 non-redundant RNA–ligand structures [34]. We found that the SASA can be used to characterize the ligand-induced conformational changes. The ligand-binding nucleotides are often situated in pocket regions on concave, rather than convex, surfaces of RNA, resulting in smaller exposed areas. Similarly, the analysis of the 60 non-redundant RNA–ligand structures revealed that nucleotides involved in binding exhibit lower LN values, indicating that the LN feature captures the surface geometry of pocket structures. Additionally, other studies have transformed RNA structures into networks to predict their functional expression [30,74]. Individual nucleotides are represented as network nodes in the RNA networks, while non-covalent interactions between nucleotides are depicted as network edges. Network topology features can capture global and local interaction features within pocket structures. For instance, RPflex utilizes the degree and clustering coefficient to characterize local interactions within the network, while network diameter is used to infer long-range interactions [49]. The results indicate that in pocket structures, hydrogen bonds formed within base interactions play a critical role in stabilizing RNA structures, whereas backbone interactions are essential for RNA folding. This approach offers an intuitive representation of complex 3D structures, facilitating the identification of critical interaction sites.

## 5. RNA–Ligand Interaction Prediction

**Figure 5:**
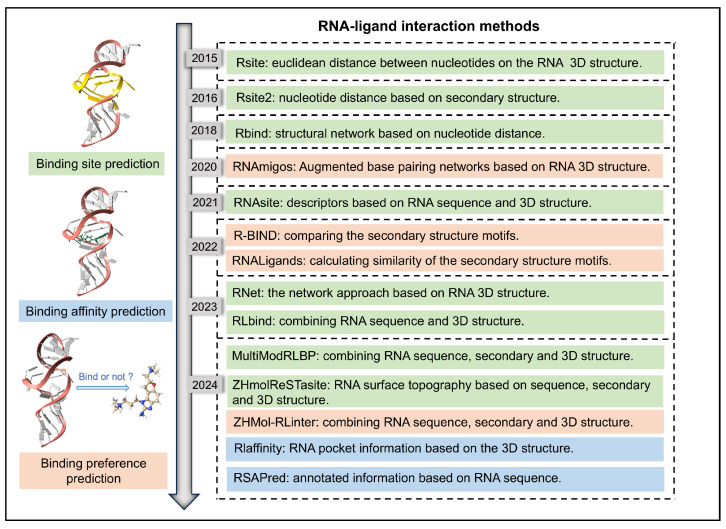
Timeline of the development of RNA–ligand interaction prediction, including binding site, binding affinity, and binding preference prediction.

By utilizing prior knowledge from experimentally determined structures, machine-learning-based methods can analyze large-scale biological data to identify complex patterns and predict previously difficult-to-detect interactions through experimental approaches [80,81,82]. RNA sequences, secondary structures, and tertiary structural features are converted into representations compatible with machine learning algorithms, such as one-hot encoding and sliding windows. When combined with embedded features of ligands, these machine learning models can predict aspects of complex interactions (Figure 5).

**Figure 6:**
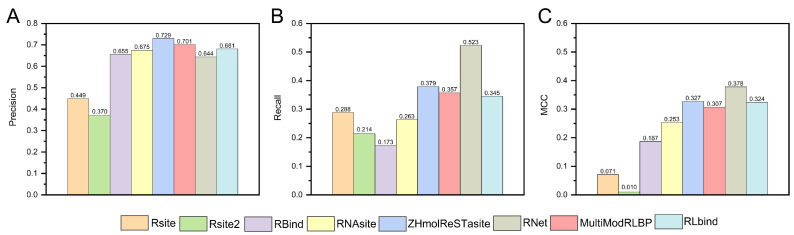
The performance of different methods for RNA–ligand binding site prediction was evaluated on the benchmark dataset TE18, including precision (**A**), recall (**B**), and Matthews correlation coefficient (MCC) (**C**).

RNA–ligand binding site prediction methods employ various RNA structural features to identify nucleotide-binding sites for ligands, typically framing the task as a binary classification problem. For example, Rsite identifies ligand-binding sites by analyzing the Euclidean distances between nucleotides within the RNA tertiary structure [83]. In contrast, Rsite2 uses secondary structures instead of tertiary ones to calculate these distances [84]. RBind and RNet convert RNA tertiary structures into structural networks to account for network attributes [30,74]. RNAsite combines features from RNA sequences and tertiary structures, including SASA, LN, and network properties, using the random forest algorithm for predicting ligand-binding sites [31]. Building upon this approach, RLbind integrates RNA sequence and tertiary structure features (network properties and SASA), along with biochemical properties, into a convolutional neural network model to capture RNA features effectively [85]. MultiModRLBP employs four modular deep learning components to incorporate RNA sequence, secondary structure, and tertiary structure features [35]. In our previous research, ZHmolReSTasite utilized a deep residual network to extract RNA sequence, secondary structure, and tertiary structure features (network properties, SASA, LN, and pocket features) for predicting ligand-binding nucleotides [34]. The performance of these methods was evaluated on the benchmark dataset TE18 (Figure 6) [74]. Results indicated that MultiModRLBP outperformed the other methods in terms of recall and Matthews correlation coefficient (MCC) metrics, while ZHmolReSTasite achieved the highest precision. RNet achieves the second-highest accuracy despite relying exclusively on network properties. ZHmolReSTasite also analyzed the role of pocket features, revealing that small molecules tend to bind within RNA pockets rather than on more convex surfaces. These results highlight that integrating RNA sequence and structural features can more accurately and comprehensively capture the specificity of RNA in recognizing ligands, especially the key role of tertiary structure geometric features.

RNA–ligand binding preference prediction methods aim to identify potential ligands that interact with RNA, offering valuable insights for functional regulation and drug development. For instance, R-BIND and RLigands utilize RNA sequence and secondary structure features to determine the specificity of small molecules [43,45]. These methods screen their databases by calculating the similarity of secondary structure motifs and predicting ligands that correspond to the target RNA. RNAmigos transforms RNA tertiary structures into networks, considering both canonical and non-canonical base-pairing interactions to predict ligand fingerprints for the target RNA [86]. However, R-BIND and RLigands overlook the characteristics of ligands, which limits their capacity to predict novel ligands. Our work, ZHMol-RLinter, focuses on learning from the RNA–ligand interaction interface. It incorporates RNA sequence, secondary structure, tertiary structure, and physicochemical environment features to predict whether a target RNA and ligand will bind [87]. Compared to other methods, ZHMol-RLinter captures interaction information by learning comprehensive features from both RNA and ligands.

**Figure 7:**
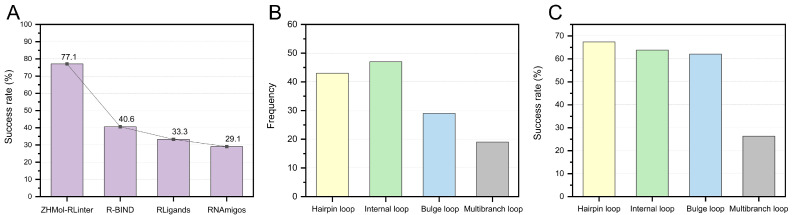
(**A**) The performance of ZHMol-RLinter, R-BIND, RLigands, and RNAmigos on the challenging UNK96 testing set. (**B**) The distribution of motif types in the UNK96 testing set, including hairpin loops, internal loops, bulge loops, and multibranch loops. (**C**) The performance of ZHMol-RLinter, R-BIND, RLigands, and RNAmigos on different loop motifs within the UNK96 testing set.

We compared the performance of R-BIND, RLigands, RNAmigos, and ZHMol-RLinter on the challenging unknown ligands in the UNK96 testing set (Table S1). As shown in Figure 7A, ZHMol-RLinter achieved a success rate of 77.1%, while the success rates for the top 20 predicted ligands in R-BIND, RLigands, and RNAmigos were 40.6%, 33.3%, and 29.2%, respectively (Table S2). This demonstrates that ZHMol-RLinter has higher accuracy and comprehensive capabilities. ZHMol-RLinter achieved an F1 score of 70.4% on predicted structures with an RMSD accuracy of 8 Å [87]. The results indicated that ZHMol-RLinter is a reliable method with notable robustness. The accuracy of predicted RNA 3D structures influences the performance of RNA–ligand binding preference predictions. Therefore, advancing RNA 3D structure prediction methods plays a crucial role in deepening our understanding of RNA–ligand interactions [88,89,90,91]. We also evaluated the contribution of different feature types in the random forest model of ZHMol-RLinter. The results indicated that structural geometric features, specifically network properties and LN, are critical in ligand-specific recognition (Figure S1).

Additionally, we assessed the performance of ZHMol-RLinter on various secondary structure motifs within the UNK96 testing set (Figure S2 and Table S3). Using a 4 Å cutoff, we identified all loop motifs in the UNK96 testing set that physically interact with ligands. This resulted in a total of 138 loop motif–ligand pairs, which included 43 hairpin loops, 47 internal loops, 29 bulge loops, and 19 multibranch loops (Figure 7B). The results showed that the success rates for hairpin, internal, and bulge loops were 67.4%, 63.8%, and 62.1%, respectively (Figure 7C). In contrast, the success rate for multibranch loops was significantly lower at only 26.3%. Multibranch loops are complex structural domains that typically consist of more nucleotides than other types of motifs. This structural complexity arises from the extensive interactions between nucleotides within multibranch loops, which facilitate the formation of intricate tertiary structures in RNA. Consequently, predicting ligands in the multibranch loop regions of RNA is particularly challenging. This limitation highlights the need for substantial improvements in current methods, especially those targeting multibranch loops, to enhance the accuracy of RNA–ligand interaction predictions.

## 6. Future Directions

RNAs stabilize the complex structures and execute their biological functions through interactions with other molecules, such as small molecular ligands and ions [92,93]. The non-specific electrostatic force between RNA and ions leads to the accumulation of ions around the RNA, resulting in significant ion–ion correlations and fluctuation effects [94]. Ion concentration can influence the RNA recognition process [95,96]. Compared to small molecular ligands, ions are nearly spherical and much smaller in size. This characteristic causes ions to distribute more diffusely on the RNA surface, resulting in non-specific binding. Unlike ion interaction mechanisms, small molecular ligands recognize specific structural regions of RNA, interacting with RNA pockets in a “key and lock” mode. Thus, our review focuses on exploring RNA–small molecule interactions, revealing how RNA sequence and structural features facilitate the specific recognition of small molecules.

RNAs interact with small molecules through binding pockets [97,98,99,100]. For example, the flavin mononucleotide (FMN) riboswitch regulates gene expression by binding the FMN ligand [101]. This binding process induces changes in the RNA secondary structures, forming stacking interactions within the binding pocket to stabilize the complex. In recent years, we have conducted detailed studies on pocket structures to unveil the mechanisms by which they recognize other molecules. We have developed the RPocket and RPpockets databases, which provide information on RNA–ligand and RNA–protein interactions with pocket topology, respectively [47,102]. To describe the geometry of RNA pockets, we have developed pocket-shape descriptors that categorize pockets as sphere-like, disc-like, or rod-like. Furthermore, we have systematically analyzed the non-catalytic pockets and constructed the HKPocket database to provide druggability information on kinase pockets, thereby advancing our understanding of regulation mechanisms [103,104]. Previous studies have shown that ligand-binding RNA pockets occupy a similar property space to protein pockets that bind ligands [105]. However, RNA–ligand interaction predictions are still inadequate compared to proteins. Thus, determining how to capture the RNA pocket features to understand the RNA–ligand interactions will be a key focus of future work.

We developed computational methods, ZHmolReSTasite and ZHMol-RLinter, utilizing SASA, LN, and network topological descriptors to characterize the geometric features of RNA pockets, effectively demonstrating their ability to specifically recognize ligands. For instance, the average Laplacian norm per nucleotide in the internal loop of the ribosomal RNA (PDB ID: 7JJU_A) is lower than that of the other two loops (Figure S3A). This suggests that the nucleotides in the internal loop are situated in the concave regions of the RNA surface, creating a pocket suitable for GZ6 ligand binding. ZHMol-RLinter accurately predicted the binding preferences of this internal loop. However, the hairpin loop is less likely to form a pocket suitable for CAC ligand binding, which contributes to ZHMol-RLinter’s inaccuracy. This may be due to the more long-range interactions present at the hairpin loop, such as the U24–A32, U25–A29, A7–G26, and G8–C27 interaction pairs. This highlights the significance of considering both local and long-range interactions in RNA–ligand interaction predictions, especially the long-distance communication information encoded by nucleotides. Additionally, ZHMol-RLinter failed to predict two unpaired single-stranded ribosomal RNA–ligand complexes, which have their 3D structures exposed to the solvent (Figure S3B). This exposure hinders the formation of RNA pockets. Therefore, investigating the interactions of these complex RNA–ligand structures could be a promising direction for the future. Compared to the advancements in RNA–ligand binding site and preference prediction, methods for predicting binding affinity are still very limited, with only two recently developed approaches (RLaffinity and RSAPred) [36,37]. Future work should comprehensively consider both RNA sequence and structural features, particularly the geometric characteristics of binding pockets. These multidimensional approaches will help more accurately capture the specificity of RNA in recognizing ligands, thereby improving the predictive performance for binding affinity.

Additionally, a notable challenge in the development of computational methods lies in the scarcity of experimental data. In the PDB database, experimentally determined RNA-related complexes account for only about 4% [23]. Even more limited are experimentally determined RNA-binding affinity data, with only 149 nucleic acid–ligand complexes included in the PDBbind 2020 version [41]. Therefore, constructing pre-trained models to enhance the training data for machine learning models represents a promising pathway for future development. For example, RLaffinity employs a self-supervised pre-training process on RNA–ligand structures without binding affinity data, aiming to maximize embedding learning from the limited data [36]. Pre-training models provide a valuable strategy for RNA interaction predictions by learning universal representations of RNA from large datasets, capturing the intrinsic patterns and structural information without relying on costly experimental annotations. Furthermore, exploring ensemble learning techniques may offer a way to combine multiple models for better prediction accuracy. For example, WVDL uses a weighted voting method based on RNA sequence information to combine convolutional neural networks, long short-term memory networks, and residual networks for protein binding site prediction [106].

This review examines advancements in the interactions between RNA and ligands, focusing on the mechanisms of these interactions and the prediction methods. Long-range forces play a key role in the recognition of RNA by ligands, while short-range forces help optimize and stabilize the complex structure. We analyze RNA–ligand interaction mechanisms from various perspectives, including sequences and structures, as well as their applications in predicting these interactions. However, the development of methods to predict binding affinity lags in the prediction of RNA–ligand binding sites and preferences, remaining in the early stages. To improve binding affinity predictions, it is crucial to integrate multidimensional RNA sequences and structural features, particularly the geometric characteristics of binding pockets. This approach can better capture the specificity of RNA in recognizing ligands and offers promising insights for future research. Additionally, utilizing pre-trained models and ensemble learning algorithms can enhance the training data for machine learning models, helping to address the limitations of experimental data. As computational methods advance, we anticipate significant breakthroughs in RNA–ligand complex research, which will lay a stronger foundation for RNA-targeted drug design and therapeutic strategies.

## Figures and Tables

**Table 1:** List of RNA–ligand databases, including time, description, data availability, and reference.

Database	Time	Description	Data Availability	Reference
SMMRNA	2014	The database of small molecule modulators along with their target RNA and experimentally determined binding data.	N/A	[14]
NALDB	2016	The database provides detailed information about the experimental data of small molecules that were reported to target all types of nucleic acid structures.	https://www.iiti.ac.in/people/~amitk/bsbe/naldb/HOME.php (9 December 2024)	[15]
Inforna 2.0	2017	A small molecule design platform for structured RNAs that integrates all known RNA motif–small molecule binding partners reported in the scientific literature.	N/A	[44]
NoncoRNA	2020	A manually curated database of ncRNAs and drug target associations designed to provide a potential resource of high-quality data for the exploration of drug sensitivity-related ncRNAs in a variety of human cancers.	http://www.ncdtcdb.cn:8080/NoncoRNA/ (9 December 2024)	[39]
RPocket	2021	An intuitive database of RNA pocket topology information with RNA–ligand data resources that provides geometrical size, centroid, shape, and secondary structure elements of RNA pockets.	http://zhaoserver.com.cn/RPocket/RPocket.html (9 December 2024)	[47]
R-BIND	2022	An updated database of bioactive RNA-targeting small molecules and associated RNA secondary structures.	https://rbind.chem.duke.edu (9 December 2024)	[45]
RNALigands	2022	A database of RNA secondary structure motifs and small molecular ligands.	https://github.com/SaisaiSun/RNALigands (9 December 2024)	[43]
HARIBOSS	2022	A database on the pocket structure and physicochemical properties of ligands and RNA.	http://hariboss.pasteur.cloud (9 December 2024)	[46]
R-SIM	2023	An experimentally validated database of RNA–small molecule interactions that provides comprehensive information on the sequence, structure, and classification of RNA, various physicochemical properties of small molecules, binding affinities, and the literature sources of the data.	https://web.iitm.ac.in/bioinfo2/R_SIM/index.html (9 December 2024)	[42]
ROBIN	2023	A library of nucleic acid binders was identified by small molecule microarray (SMM) screening that reported 2003 RNA–ligand small molecules, representing the largest fully publicly available experimentally derived library to date.	https://github.com/ky66/ROBIN (9 December 2024)	[40]

## Supplementary Materials

The following supporting information can be downloaded at: https://www.mdpi.com/article/10.3390/life15010104/s1, Figure S1. The importance of different features in the Random Forest model of ZHMol-RLinter. GE represents the geometric features, including LN and network properties; Figure S2. Example of RNA secondary structural motifs from the Arabidopsis thaliana thiamine pyrophosphate riboswitch with its regulatory ligand (PDB ID: 2CKY), including multibranch loop, bulge loop, internal loop, and hairpin loop; Figure S3. (A) The interaction of ribosomal RNA–ligand complexes (PDB ID:7JJU_A). The ribosomal RNA includes a hairpin loop, a bulge, and an internal loop. The hairpin loop has one hydrogen bond and five hydrophobic contacts with ligand (CAC). The internal loop has two hydrogen bonds and four hydrophobic contacts with ligand (GZ6). (B) The unpaired single-stranded ribosomal RNA–ligand complexes; Table S1. List of RNA and small molecules in the UNK96 testing set for ZHMol-RLinter; Table S2. The success rates of ZHMol-RLinter, RLigands, RNAmigos, and R-BIND in the UNK96 testing set; Table S3. The success rates of ZHMol-RLinter in different loop types in the UNK96 testing set.
