# Supplementary material for: Advances and Mechanisms of RNA–Ligand Interaction Predictions
Source: Life (Basel). 2025 Jan 15;15(1):104. doi: 10.3390/life15010104 (PMC11767038; doi:10.3390/life15010104)
Supplement: Supplementary file 1 [file life-15-00104-s001.zip › life-3390742-supplementary.pdf]

Supplementary materials

Advances and mechanisms of RNA-ligand interaction predictions

Chen Zhuo[1], Chengwei Zeng[1], Haoquan Liu[1], Huiwen Wang[2], Yunhui Peng[1] and Yunjie Zhao[1]*

[1]Institute of Biophysics and Department of Physics, Central China Normal University, Wuhan, 430079, China

[2]School of Physics and Engineering, Henan University of Science and Technology, Luoyang 471023, China

* Correspondence: yjzhaowh@ccnu.edu.cn

Figures and tables

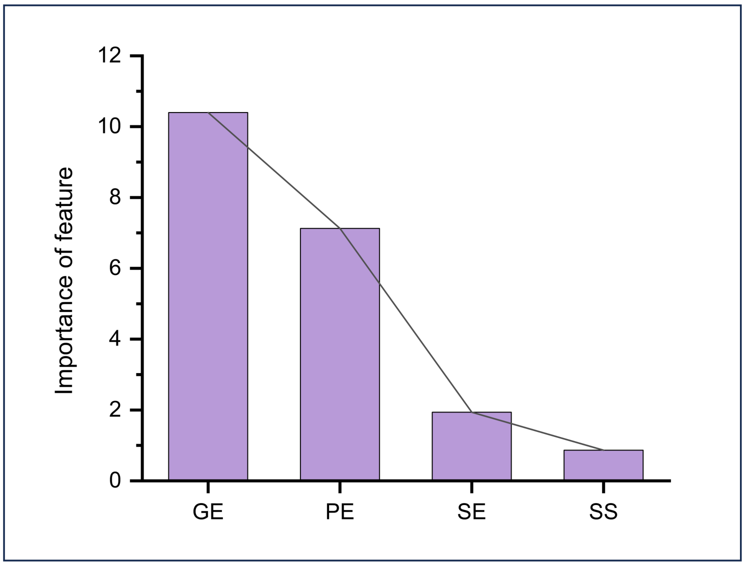

Figure S1. The importance of different features in the Random Forest model of ZHMol-RLinter. GE represents the geometric features, including LN and network properties. PE represents the physicochemical environment feature. SE represents the sequence feature. SS represents the secondary structural feature.

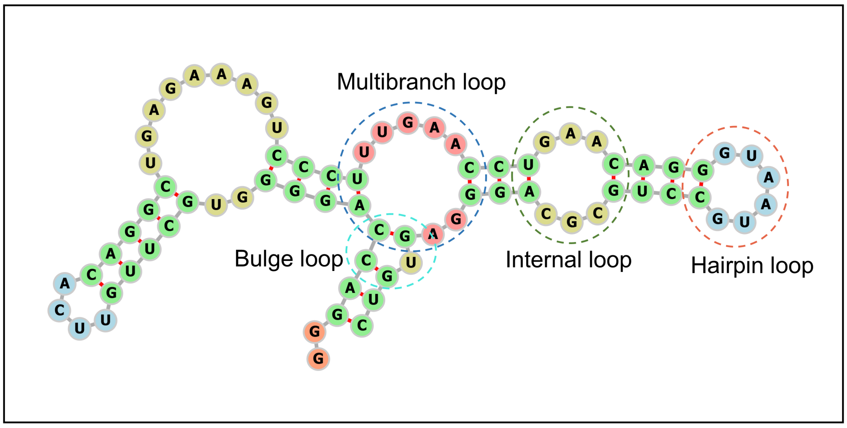

Figure S2. Example of RNA secondary structural motifs from the Arabidopsis thaliana thiamine pyrophosphate riboswitch with its regulatory ligand (PDB ID: 2CKY), including multibranch loop, bulge loop, internal loop, and hairpin loop.

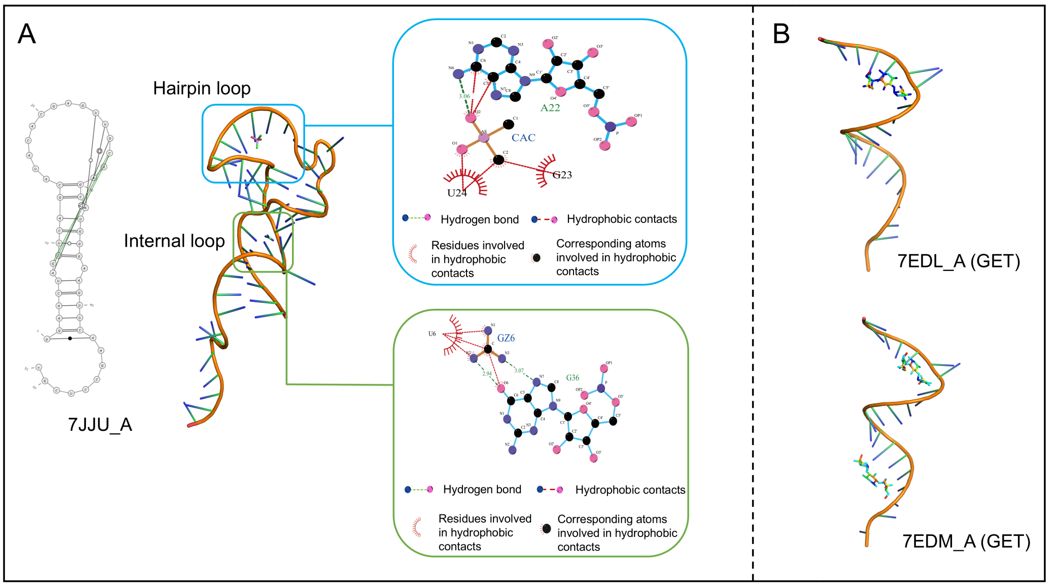

Figure S3. (A) The interaction of ribosomal RNA-ligand complexes (PDB ID:7JJU_A). The ribosomal RNA includes a hairpin loop, a bulge, and an internal loop. The hairpin loop has one hydrogen bond and five hydrophobic contacts with ligand (CAC). The internal loop has two hydrogen bonds and four hydrophobic contacts with ligand (GZ6). (B) The unpaired single-stranded ribosomal RNA-ligand complexes.

Table S1. List of RNA and small molecules in the UNK96 testing set for ZHMol-RLinter.

PDB ID_chain	Small molecule
6TF0_A	NAI
6TF1_A	ADP
6TF2_A	ATP
6TF3_A	3AT
6TFE_A	N6E
6TFF_A	NAD
6TFG_A	PPS
6U8F_A	TG
6UP0_C	YO3
6WZR_A	UG1
6WZS_A	UG4
6XB7_A	UYS
6XRQ_A	V8A
6Y3G_F	GAI
6Y3G_F	GOL
7D7W_A	NAD
7D7X_A	ADP
7D7Y_A	ATP
7D82_A	NAD
7E9I_A	J0C
7EDL_A	J0C
7EDM_A	J0C
7EDT_A	SPM
7ELP_A	XAN
7ELS_A	AZA
7EOH_A	J8F
7EOK_A	J8L
7EOL_A	J8O
7EOM_A	J8R
7EON_A	J8U
7EOO_A	J8X
7EOP_A	J93
7FHI_A	53D
7FJ0_A	53D
7JJU_A	CAC
7JJU_A	GZ6
7KVT_B	2ZY
7L0Z_G	2ZY
7L0Z_G	SPM
7MKT_A	MMP
7MLW_F	GAI
7OAW_A	V6T

7OAW_C	SPM
7OAX_A	GOL
7OAX_A	V5Z
7QP2_A	GOL
7QSH_A	HYJ
7SXP_A	CAD
7TD7_A	VIB
7TDB_A	GMI
7TZR_X	KWU
7TZS_X	KX3
7TZT_A	KXC
7TZU_A	KWL
7V9E_A	GUN
7WI9_V	SEY
7WI9_V	THE
7WIE_V	7DG
7WIF_V	H4B
7WII-V	NPR
7XD6_N	SPD
7ZJ4_E	J93
8CF2_A	ULR
8D2A_A	QB3
8D2B_A	QAX
8D5L_A	QIJ
8D5O_A	QEU
8EYU_A	X5R
8EYW_B	TFX
8F4O_B	PG4
8F4O_B	PGE
8HB3_A	NNR
8HB3_B	NNR
8HB8_A	NMN
8HBA_A	NAD
8I3Z_B	NMN
8I43_A	53D
8I44_A	53D
8I45_A	53D
8I46_A	53D
8I7N_N	OJI
8R63_B	Y53
8R8P_A	YB3
8R8P_B	YB3
8SWG_A	G3A
8SWO_A	G3A

8SX5_A	G3A
8TQX_D	GOL
8TSV_C	GOL
8U5J_A	DMS
8U5J_A	W6F
8U5K_A	VK0
8U5P_A	VKI
8U5T_A	VLR
8U5Z_B	W6F
8VAW_A	DGP

Table S2. The success rates of ZHMol-RLinter, RLigands, RNAmigos, and R-BIND in the UNK96 testing set.

	ZHMol-RLinter	RLigands			RNAmigos			R-BIND		
		Top1	Top10	Top20	Top1	Top10	Top20	Top1	Top10	Top20
T[1]	74	3	22	32	3	15	28	3	25	39
F[2]	22	93	74	64	93	85	68	93	71	57
Success rate[3]	77.1%	3.1%	22.9%	33.3%	3.1%	15.6%	29.2%	3.1%	26.0%	40.6%

[1]T indicates the number of RNA-ligand complexes correctly predicted the target ligand or binding preference; [2]F shows the number of RNA-ligand complexes incorrectly predicted the target ligand or binding preference; [3]Success rate: T/(T+F).

Table S3. The success rates of ZHMol-RLinter in different loop types in the UNK96 testing set.

	Hairpin loop	Internal loop	Bulge loop	Multibranch loop
T[1]	29	30	18	5
F[2]	14	17	11	14
Success rate[3]	67.4%	63.8%	62.1%	26.3%

[1]T indicates the number of RNA-ligand complexes that correctly predicted the binding preference; [2]F shows the number of RNA-ligand complexes that incorrectly predicted the binding preference; [3]Success rate: T/(T+F).
